# Chimeric Tobamoviruses With Coat Protein Exchanges Modulate Symptom Expression and Defence Responses in *Nicotiana tabacum*

**DOI:** 10.3389/fmicb.2020.587005

**Published:** 2020-11-06

**Authors:** Man Yu, Xinyue Bi, Yuanmin Huang, Yong Chen, Jun Wang, Ruina Zhang, Yunkang Lei, Zihao Xia, Mengnan An, Yuanhua Wu

**Affiliations:** ^1^Liaoning Key Laboratory of Plant Pathology, College of Plant Protection, Shenyang Agricultural University, Shenyang, China; ^2^Sichuan Tobacco Company Deyang City Company, Deyang, China

**Keywords:** tobacco mosaic virus, coat protein, transcriptome analysis, chimeric viruses, plant hormones

## Abstract

In the pathogen infection and host defence equilibrium, plant viruses have evolved to efficiently replicate their genomes, to resist the attack from host defence responses and to avoid causing severe negative effect on growth and metabolism of the hosts. In this study, we generated chimeric tobacco mosaic virus (TMV) variants, in which the coat protein (CP) sequences were substituted with that of cucumber green mottle mosaic virus (CGMMV) or pepper mild mottle virus (PMMoV) to address the role of these in virus infection and host symptomology. The results showed that the chimeric viruses (TMV-CGCP or TMV-PMCP) induce stunting and necrotic symptoms in tobacco plants. We analyzed the transcriptomic changes in tobacco plants after infection of TMV and its chimeras using a high-throughput RNA sequencing approach and found that infection of the chimeric TMV induced significant up-regulation of host defence responsive genes together with salicylic (SA) or abscisic acid (ABA) responsive genes, but down-regulation of auxin (Aux) responsive genes. We further confirmed the increase in the levels of SA and ABA, together with the reduced levels of Aux after infection of chimeric TMV in tobacco plants. These data suggest novel roles of tobamovirus CP in induction of host symptoms and defence responses.

## Introduction

Plant viruses are obligate intracellular parasites and rely on the host machinery for the establishment of their infection cycle (McLeish et al., 2018). Virus-host interaction induces significant molecular alterations in the host plant, which leads to the display of disease symptoms (Lu et al., 2012). Plants usually develop symptoms such as mosaic, stunting, chlorosis or necrosis after virus infection, which may significantly reduce the quality of crops (McLeish et al., 2018; Conti et al., 2017). To combat virus infection, plants mainly employ innate immunity such as pathogen-associated molecular pattern (PAMP)-triggered immunity (PTI) and effector-triggered immunity (ETI) (Gouveia et al., 2016; Calil and Fontes, 2017). In addition, plants have developed RNA silencing, autophagy or other strategies to limit the virus (Ding, 2010; Kushwaha et al., 2019). To counteract host defence, plant viruses also use elaborate ways to suppress or evade host defence machinery to ensure their survival (Wang, 2015; Pallas and Garcia, 2011). In this process, plant viruses hijack host factors for their genome replication, intercellular movement and systemic infection, leading to significant alterations in host gene regulatory network (e.g., genes involved in defence responses and multiple signaling cascades) and metabolic pathway (e.g., biosynthesis of various phytohormones) (Whitham et al., 2003; Collum and Culver, 2016). The high-throughput sequencing techniques, such as RNA sequencing (RNA-Seq) are powerful tools for the identifications of genes whose expression is altered during virus infection in plants (Chen et al., 2017; Li et al., 2017; Sheng et al., 2019).

Tobacco mosaic virus (TMV) belongs to the genus *Tobamovirus* with a positive single-strand RNA and the genome encodes at least four proteins (Rybicki, 2015). Among them, the 126 and 183 kDa proteins are translated from TMV genomic RNA and play crucial roles in viral genome replication (Ishibashi and Ishikawa, 2016). The 30 kDa movement protein (MP) expressed from sub-genomic RNA facilitates movement of the virus between host cells. The 17.5 kDa coat protein (CP) of TMV is a multifunctional protein responsible for virion formation, viral systemic movement, cross-protection and genomic RNA stability (Callaway et al., 2001; Ivanov and Mäkinen, 2012). It was reported that the sequences of TMV CP are responsible for the differential disease symptomology of the infected plants (Dawson et al., 1988; Culver, 2002). Furthermore, viral CP was also shown to contribute significantly to determining host range of tobamoviruses (Weber and Bujarski, 2015).

The researches of TMV and other tobamovirus species have greatly contributed to the development of virology and viral evolution (Moury et al., 2017). Viruses like cucumber green mottle mosaic virus (CGMMV) and pepper mild mottle virus (PMMoV) are well-investigated tobamoviruses and their genetic structures are similar to that of TMV (Dardick et al., 1999), but only have approximately 45–65% nucleotide sequence similarity. For example, certain viral species have evolved the differential host ranges in which CGMMV mainly infects the family Cucurbitaceae while PMMoV infects pepper plants (Li et al., 2015; Dombrovsky et al., 2017). In contrast, TMV seems to have wider host range than that of CGMMV and PMMoV. To date, the critical viral proteins or the nucleotide sequences involved in the determination of host ranges of tobamoviruses have not been well understood.

The application of homologous recombination technology to construct chimeric viruses provide important tools to unravel the functions of specific viral RNA sequences or the coding proteins during infection (Yelina et al., 2002; Lukhovitskaya et al., 2013; Tuo et al., 2015; Yu et al., 2019). In a previous study, we generated a PMMoV infectious clone and several chimeric clones to clarify critical RNA sequences required for efficient virus infection (Yu et al., 2019).

In this study, we constructed two chimera TMV-CGCP and TMV-PMCP, in which the CP of TMV was substituted with that of CGMMV and PMMoV using the homologous recombination technique. Intriguingly, *Nicotiana tabacum* inoculated with the chimeric viruses showed significant stunting compared with those inoculated with wild-type TMV. We further investigated the global changes of gene expressions during TMV and the chimeric virus infection in *N. tabacum* using RNA-seq technology. The results showed that the host resistance genes were significantly up-regulated in leaves infected with TMV-CGCP or TMV-PMCP. Furthermore, infection of the chimeras also induced up-regulation of the genes involved in host defence responses and affected phytohormone biosynthesis or signaling. Specifically, the levels of salicylic acid (SA) and abscisic acid (ABA) were increased, whereas the levels of auxin was decreased by infection of the chimeric viruses.

## Materials and Methods

### Construction of pCB-TMV-CGCP and pCB-TMV-PMCP

**FIGURE 1:**
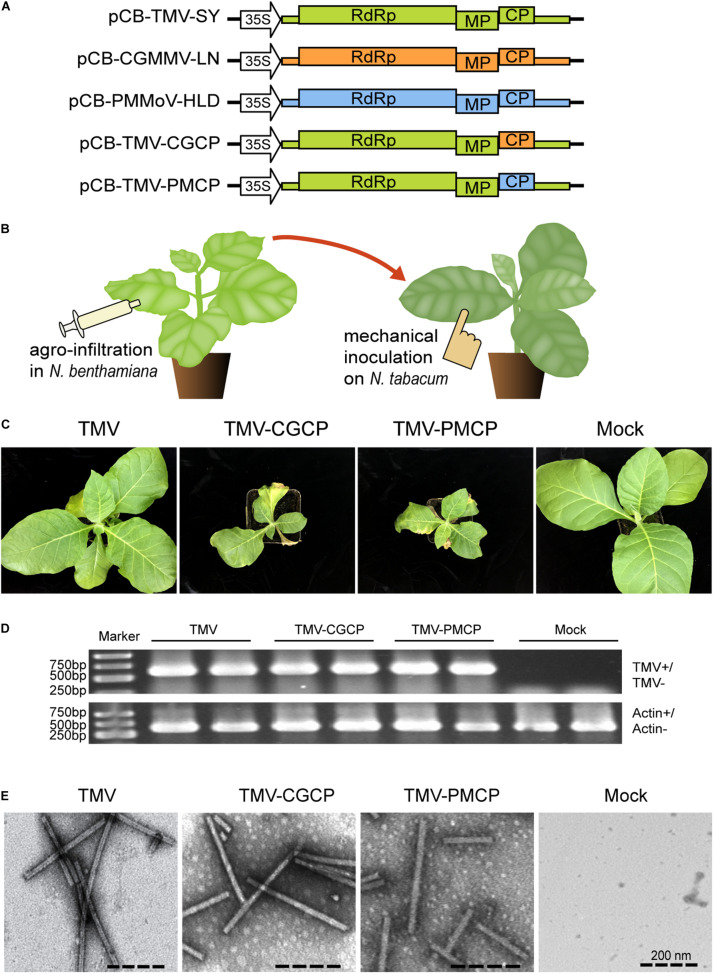
Symptoms of *N. tabacum* infected by TMV or the chimeric viruses TMV-CGCP, TMV-PMCP. Mock-inoculated plants were used as control treatments (Mock). **(A)** Schematic representation of the TMV, CGMMV, PMMoV and the chimeras of TMV constructed in this study. **(B)** Diagram of the virus inoculation methods. *Agrobacterium tumefaciens* strain GV3101 containing pCB-TMV-SY, pCB-TMV-CGCP or pCB-TMV-PMCP was infiltrated into leaves of *N. benthamiana* and then the sap of its upper leaves were mechanically inoculated onto leaves of *N. tabacum*. **(C)** Symptoms developed by TMV, TMV-CGCP and TMV-PMCP on *N. tabacum*. Infection of TMV-CGCP or TMV-PMCP induced significant stunting and necrotic symptoms in *N. tabacum* compared with those infected by wild-type TMV at 12 dpi. **(D)** RT-PCR analysis of TMV and the chimeras from the systemic leaves of *N. tabacum*. Host gene *actin* was used as a control. **(E)** Electron micrograph of viral particles of TMV and the chimeras isolated from *N. tabacum* leaves.

To construct pCB-TMV-CGCP and pCB-TMV-PMCP that express chimeric TMV encoding heterologous coat protein sequences from CGMMV and PMMoV (Figure 1A), the plasmid pCB-TMV-SY, an infectious clone of TMV Shenyang isolate (MG516107) was used as a template, and TMV-CG + /TMV-CG- and TMV-PM + /TMV-PM- primer pairs were used to amplify two vector DNA fragments with approximately 1,1000 bp in size by TransStart FastPfu PCR SuperMix (TransGen Biotech, Beijing, China). The amplified PCR products were thereafter treated with DMT enzyme (TransGen, Biotech, China) to digest the non-mutated parental plasmid. Two insert DNA fragments were amplified using pCB-CGMMV-LN (KY040049) and pCB-PMMoV-HLD (Yu et al., 2019; Figure 1A) as templates by primer pairs T-CG-CP + /T-CG-CP- and T-PM-CP + /T-PM- CP-, respectively, using high fidelity KOD Plus DNA polymerase (Toyobo, Osaka, Japan). Then the PCR products were purified using an agarose gel DNA Recovery Kit (TIANGEN, Beijing) and ligated with the amplified vector fragments using ClonExpressTM MultiS One Step Cloning Kit (Vazyme, Nanjing, China).

### Construction of pGD-TMCP-Myc, pGD-CGCP-Myc and pGD-PMCP-Myc

Plasmids of pCB-TMV-SY, pCB-CGMMV-LN and pCB-PMMoV-HLD were used as templates to generate 510 bp, 516 bp and 504 bp sized PCR products using primer pairs TMV-CP + /TMV-Myc- CP-, PMMoV-CP + /PMMoV-Myc-CP- and CGMMV-CP + /CGMMV-Myc- CP-, respectively. The three amplified DNA fragments were integrated to the BgIII and *Hin*dIII double digested pGD vector using ClonExpressTM MultiS One Step Cloning Kit (TransGen, Biotech, China), respectively, to construct pGD-TMCP-Myc, pGD-CGCP-Myc and pGD-PMCP-Myc.

### Construction of pCB-TMV-fsCP, pCB-TMV-fsCGCP and pCB-TMV-fsPMCP

To construct plasmids of pCB-TMV-fsCP, pCB-TMV-CGfsCP and pCB-TMV-PMfsCP (Supplementary Figure S1A) that cannot express the respective viral coat proteins, pCB-TMV-SY, pCB-CGMMV-LN and pCB-PMMoV-HLD were used as templates and were amplified by primer pairs TMVfsCP + /TMVfsCP-, T-CGCP-fsCP + /T-CGCP- fsCP-, T-PMCP-fsCP + /T-PMCP- fsCP-, respectively. The amplified DNA fragments were treated with DMT enzymes and subjected to self-ligation using a Fast Mutagenesis System kit (TransGen, Biotech, China). The constructs were transformed into *Agrobacterium tumefaciens* GV3101. All of the PCR primers used in this study are listed in Supplementary Table S1.

### Virion Purification and Microscopic Observation

The virions of wild-type TMV, TMV-CGCP, and TMV-PMCP were extracted and purified from the corresponding *agrobacterium* infiltrated *N. benthamiana* leaves using Gooding’s method (Gooding and Hebert, 1967). The purified virions adjusted to the final concentration of 2 mg/ml were treated by phosphotungstic acid stain method (Quintarelli et al., 1971) and were observed using a transmission electron microscopy HT7700 Exalens (Hitachi, Ltd., Tokyo, Japan).

### Virus Inoculation in Plants

Plants of *N. benthamiana* and *N. tabacum* cv. NC89 were cultivated in a growth climate chamber at 25°C with 16 h/8 h (light/dark) cycles. *A. tumefaciens* cultures harboring pCB-TMV, pCB-TMV-CGCP or pCB-TMV-PMCP were infiltrated into *N. benthamiana* leaves at (5–7) leaf stage. The upper leaves of *N. benthamiana* infected with TMV, TMV-CGCP or TMV-PMCP at 7 days post inoculation (dpi) were harvested and mechanically inoculated on the leaves of *N. tabacum* variety NC89 for symptom observation and subsequent analysis.

### RNA Extraction, cDNA Library Construction and Illumina Sequencing

Total RNA was extracted from leaves of *N. tabacum* that were inoculated with wild-type or chimeric TMV using TRIzon Reagent (TIANGEN, Beijing, China). Three independent biological replicates were used for RNA-seq. Sequencing libraries were generated using NEBNext UltraTM RNA Library Prep Kit for Illumina (NEB, United States) according to the manufacturer’s instructions and index codes were added to attribute sequences for each sample. The mRNA was purified from total RNA using poly-T oligo-attached magnetic beads and fragmented using divalent cations at elevated temperatures in NEBNext First Strand Synthesis Reaction Buffer (5X). The cDNA was synthesized and purified with AMPure XP system (Beckman Coulter, Beverly, United Statesto select approximately 240 bp fragments. The size-selected, adaptor-ligated cDNA was treated with USER Enzyme (NEB, United States) before PCR, then the purified PCR products were submitted to sequence on an Illumina sequencing platform (HiSeq^TM^ 4000) conducted by the Biomarker Technologies Corporation, Beijing, China.

The raw reads generated by Illumina sequencing were submitted to the Sequence Read Archive database at NCBI (SRA)^1^, with the SRA BioProject accession number [PRJNA594802 (TMV-inoculated samples): SRR10662682, SRR10662681, SRR10662680; PRJNA594806 (TMV-CGCP-inoculated samples): SRR10662707, SRR10662706, SRR10662705; PRJNA594848 (TMV-PMCP-inoculated samples): SRR10664114, SRR10664113, SRR10664112; PRJNA594821 (Mock-inoculated samples): SRR10662770, SRR10662769, SRR10662768]. The clean reads were mapped to the reference genome sequence using Hisat2 tools soft.^2^ Differential expression analysis of TMV vs. TMV-CGCP and TMV vs. TMV-PMCP were performed using the DEseq.^3^ The differentially expressed genes (DEGs) were determined by adjusting *P* values to <0.01 using the Benjamini and Hochberg’s approach for controlling the false discovery rate <0.05. To further analysis of DEGs, Gene Ontology (GO) and Kyoto Encyclopedia of Genes and Genomes (KEGG) pathway^4^ were implemented.

### Phytohormone Analysis

Plant salicylic acid (SA), jasmonic acid (JA), abscisic acid (ABA), and indole-3-acetic acid (IAA) were determined as described with some modifications (Pan et al., 2010). The leaves of *N. tabacum* inoculated with TMV, TMV-CGCP, TMV-PMCP or buffer control were ground into powder with liquid nitrogen. Approximately 50 mg sample powder were weighed and mixed with working internal standard solution. Then plant hormones were extracted with extraction solvent (Isopropyl alcohol: water: hydrochloric acid = 2:1:0.002). The homogenate was incubated at 4°C on a shaker at a speed of 900 rpm for 30 min. Then trichloromethane (1 ml) was added to each sample and placed on the shaker at a speed of 900 rpm for an additional 30 min at 4°C. Two phases were formed by centrifuging at 14,000 rpm and 4°C for 5 min. Then the solvent from the lower phase (1.2 ml) was transferred to a new tube and concentrated using a nitrogen generator with nitrogen flow. The concentrated samples were resuspended in 0.1 ml methanol and centrifuged for 5 min at 14,000 rpm. Finally, the supernatants were filtered using a syringe and syringe filter and prepared for high-performance liquid chromatography-mass spectrometry (HPLC-MS) analysis.

### Real-Time Quantitative PCR

The RNA samples were reverse-transcribed to synthesize cDNA using a HiScript II Q RT SuperMix for qPCR kit (Vazyme, Nanjing, China). Real-time quantitative PCR (RT- qPCR) was performed using Universal ChamQTM Universal SYBR^®^ qPCR Master Mix (Vazyme, Nanjing, China). Three biological replications were performed for each treatment. Actin was used as a reference for the analysis of gene expression. The primers used for RT-qPCR were shown in Supplementary Table S2.

### Western Blot Analysis

The leaves of 3-week-old *N. benthamiana* infiltrated with pGD-TMCP-Myc, pGD-PMCP-Myc or pGD-CGCP-Myc were collected and grinded with plant protein extraction buffer [25 mM Tris, pH 7.5, 10% (v/v) glycerol, 150 mM NaCl, 1 × protease inhibitor cocktail (Roche), and 0.15% (v/v) Non-idet P-40]. After centrifugation at 12,000 rpm for 10 min at 4°C, the supernatant was collected. The total proteins were separated on 12% sodium dodecyl sulfate polyacrylamide gel electrophoresis (SDS-PAGE) gels and stained with Coomassie Brilliant Blue, thereafter transferred to polyvinylidene difluoride (PVDF) membranes (Millipore, Billerica, United Staes). The membrane was treated with Myc-tag antibody (Abcam, Shanghai, China) and alkaline phosphatase (AP)-conjugate goat anti-rabbit secondary antibody (Sangon Biotech, Shanghai, China). The treated membrane was incubated with chemiluminescent substrate CDP-star (Roche Mannheim, Germany) and visualized the target bands by using the Chemical luminous imaging system Tanon 5200 (Tanon, Shanghai, China).

## Results

### Infection of TMV and Its Chimeras Caused Different Symptoms in *N. tabacum*

*Nicotiana benthamiana* is well applied as a model plant in the study of plant physiology or pathology including plant virology. Especially, *N. benthamiana* is extremely sensitive to virus infection and can be systemically infected by a wide range of viruses (Bally et al., 2018). In this study, we constructed chimeric virus expression vectors to investigate the functions of three tobamovirus coat proteins on the pathogenicity of wild-type and chimeric TMV (Figure 1A). *A. tumefaciens* GV3101 containing pCB-TMV, pCB-TMV-CGCP or pCB-TMV-PMCP were infiltrated into *N. benthamiana* leaves and visible shriveled symptoms appeared on the upper non-inoculated leaves at 5 dpi. RNA extracted from upper un-inoculated leaves of 10 agro-infiltrated *N. benthamiana* plants were reverse transcribed and subjected to sequencing analysis. The results demonstrated that no sequence mutation occurred in the chimeric viruses after their systemic infection in plants (data not shown). Consequently, the leaves of *N. benthamiana* infected with the wild-type or the chimeras of TMV were extracted and mechanically inoculated onto the leaves of *N. tabacum* (Figure 1B). The results showed that TMV together with the chimeric viruses can cause chlorosis and mosaic symptoms on the upper un-inoculated leaves of *N. tabacum* at 12 dpi (Figure 1C). Interestingly, TMV-CGCP and TMV-PMCP also induced stunting in *N. tabacum* and mosaic symptoms on the upper leaves compared with those inoculated with wild-type TMV (Figure 1C). Specifically, the leaves inoculated by TMV-CGCP and TMV-PMCP typically showed necrotic symptoms (Figure 1C). Furthermore, we confirmed the systemic infection of TMV, TMV-CGCP or TMV-PMCP in *N. tabacum* by detecting viral RNA from the upper systemic leaves by RT-PCR (Figure 1D). To exclude the effect of CP on virus accumulation and host symptoms, we also further constructed three chimeric viruses TMV-fsCP, TMV-CGfsCP and TMV-PMfsCP that cannot express CP by introducing point mutation at the start codon of the respective CP ORF (Supplementary Figure S1A). The results showed that all of *N. tabacum* inoculated with the CP defective viruses did not develop observable diseased symptoms. We also confirmed their systemic infection in the upper leaves and relative RNA accumulation by RT-qPCR at 10 dpi (Supplementary Figures S1B,C). The accumulation levels of TMV were much higher than that of the chimeric viruses by RT-qPCR quantification (Supplementary Figure S1C). Furthermore, the accumulation levels of TMV, TMV-CGCP and TMV-PMCP were approximately 11-, 2-, and 3-folds higher than that of TMV-fsCP, TMV-CGfsCP and TMV-PMfsCP (Supplementary Figure S1C). The results also showed no significant differences between the accumulation of TMV-fsCP vs. TMV-CGfsCP and TMV-fsCP vs. TMV-PMfsCP.

### Virion Formation of Wild-Type and Chimeric TMV

Transmission electron microscope was used to observe the virus particles to determine whether the introduced heterologous coat protein can affect the virion morphology of the chimeric viruses. The results indicated that TMV-CGCP and TMV-PMCP can form rod-like virions with approximately 300 nm in length as well as wild-type TMV (Figure 1E) and there was no significant difference in the virion morphology between wild-type TMV and its chimeras, which indicated that the heterologous substituted CP do not affect the virion formation.

### Identification of the Critical Genes in Response to Infection of Wild-Type and Chimeric TMV by RNA-seq

**FIGURE 2:**
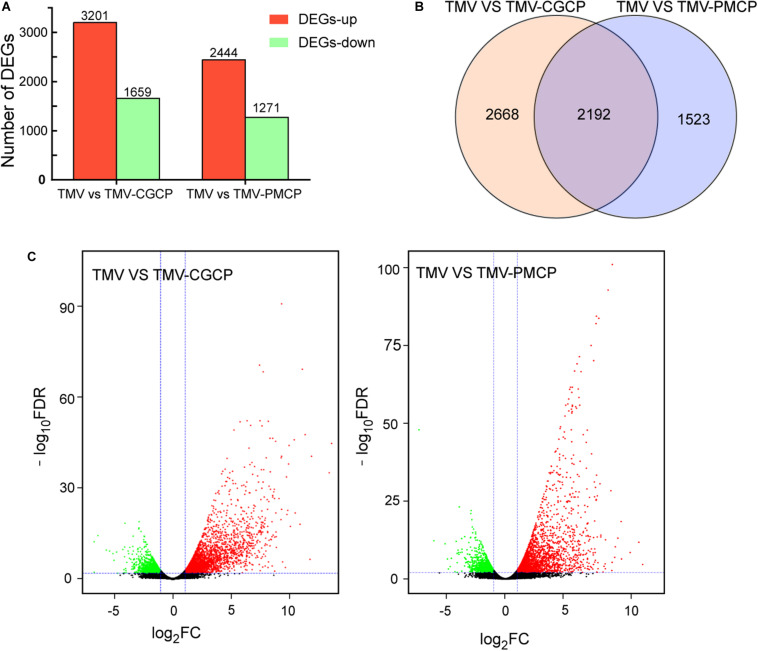
Differential gene expression in virus-infected *N. tabacum* plants. **(A)** Numbers of up-regulated (red) and down-regulated (green) gene were shown. **(B)** Venn diagram of differentially expressed genes (DEGs) between TMV vs. TMV-CGCP and TMV vs. TMV-PMCP. **(C)** Volcano plots showing DEGs of TMV vs. TMV-CGCP and TMV vs. TMV-PMCP. The red and green colors represent the significantly up- and down-regulated genes, respectively, (FDR<0.05 and |log_2_ FC| ≥ 1).

**FIGURE 3:**
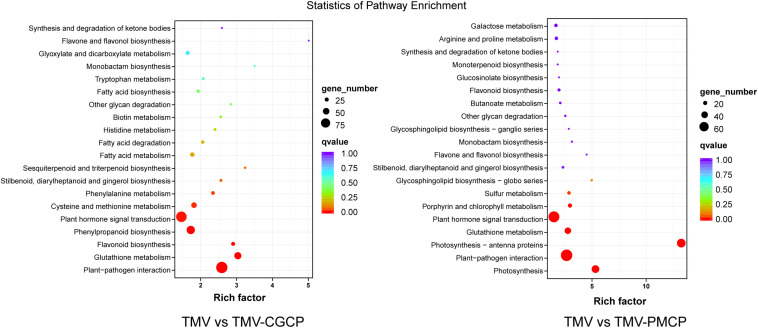
Kyoto Encyclopedia of Genes and Genomes (KEGG) pathway enrichment analysis of TMV vs. TMV-CGCP and TMV vs. TMV-PMCP. The rich factor reflects the degree of enriched DEGs in a given pathway. The number of enriched DEGs in the pathway is indicated by the circle area, and the circle color represents the ranges of the corrected *p*-value.

To further investigate the critical determinants that modulate the host symptoms among TMV and the chimeras in *N. tabacum*, genome-wide gene expression analysis was conducted and the upper systemic leaves of TMV or the chimeric viruses inoculated *N. tabacum* plants were collected for RNA-seq. The mock-inoculated plants treated with PBS only were used as negative controls and each treatment had three biological replicates. A total of 70.28 Gb raw reads were obtained and the clean reads were mapped to the reference genome database using HISAT2 software (Table 1). A total of 4860 DEGs were obtained in the TMV vs. TMV-CGCP, including 3210 up-regulated and 1659 down-regulated genes were identified. In TMV vs. TMV-CGCP, 3715 DEGs were obtained including 2444 up-regulated and 1271 down-regulated genes (Figure 2A and Supplementary Table S3). A total of 2192 DEGs in TMV vs. TMV-CGCP overlap with those in TMV vs. TMV-PMCP (Figure 2B). Differentially regulated genes were identified based on the criteria of significance FDR<0.05 and log_2_-fold change (log_2_FC) ≥ 1.0 or ≤ −1.0 (Figure 2C). The DEGs of each treatment are listed in Supplementary Table S3. To further classify the DEGs, GO and KEGG analysis were performed (Supplementary Tables S4, S5). The DEGs were mainly classified into various functions, including transporter activity, signal transducer activity and nucleic acid binding transcription factor activity in GO analysis (Supplementary Figure S4). Total annotation of the DEGs in KEGG pathways were shown (Figure 3 and Supplementary Table S6). The results indicated both TMV vs. TMV-CGCP and TMV vs. TMV-PMCP were significantly enriched in plant-pathogen interaction and plant hormone signal transduction pathways (Figure 3). Then we selected several key DEGs involved in plant-pathogen interaction and plant hormone signal transduction pathways for further investigation.

**TABLE 1:** Illumina sequencing data and the read numbers aligned onto the *N. tabacum* reference genome.

Samples	Total Reads	Mapped Reads	Uniq Mapped Reads	Multiple Map Reads	Reads Map to ‘+’	Reads Map to ‘−’
TMV-CGCP1	56208776	53,224,521 (94.69%)	51,534,374 (91.68%)	1,690,147 (3.01%)	26,239,364 (46.68%)	26,356,188 (46.89%)
TMV-CGCP2	54609134	51,633,333 (94.55%)	49,899,084 (91.37%)	1,734,249 (3.18%)	25,380,995 (46.48%)	25,522,549 (46.74%)
TMV-CGCP3	49057288	45,477,627 (92.70%)	42,913,047 (87.48%)	2,564,580 (5.23%)	21,727,533 (44.29%)	22,263,509 (45.38%)
TMV-PMCP1	54016314	50,208,864 (92.95%)	47,914,990 (88.70%)	2,293,874 (4.25%)	24,381,858 (45.14%)	24,629,293 (45.60%)
TMV-PMCP2	55549284	52,598,478 (94.69%)	50,901,575 (91.63%)	1,696,903 (3.05%)	25,914,031 (46.65%)	25,995,226 (46.80%)
TMV-PMCP3	53930648	51,747,515 (95.95%)	50,026,180 (92.76%)	1,721,335 (3.19%)	25,457,844 (47.20%)	25,560,569 (47.40%)
TMV1	46649814	44,533,379 (95.46%)	42,594,079 (91.31%)	1,939,300 (4.16%)	21,626,099 (46.36%)	21,859,544 (46.86%)
TMV2	48984922	46,923,906 (95.79%)	45,385,871 (92.65%)	1,538,035 (3.14%)	23,095,618 (47.15%)	23,195,405 (47.35%)
TMV3	51252442	49,016,576 (95.64%)	47,659,287 (92.99%)	1,357,289 (2.65%)	24,238,861 (47.29%)	24,280,600 (47.37%)

**FIGURE 4:**
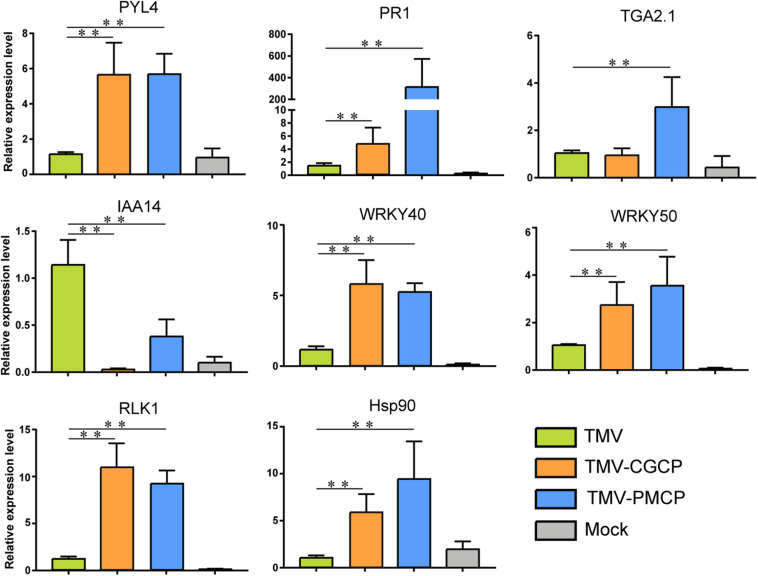
RT-qPCR analysis of the relative expression levels of *PYL4*, *IAA14*, *Hsp90*, *WRKY40*, *WRKY50*, *RLK1*, *PR1*, and *TGA2.1* in the TMV, TMV-CGCP or TMV-PMCP infected samples. Asterisks indicate a statistically significant difference compared with TMV, “^∗∗^” indicate an extremely significant difference (*P* < 0.01). Primers used for qPCR were listed in Supplementary Table S6. Information of the genes indicated in the figure were showed in Table 2. Mock-inoculated plants were used as control treatments (Mock).

To validate the results of RNA-seq, 8 genes responding to TMV or the chimeras infection in *N. tabacum* were selected for RT-qPCR verification. The selected genes included abscisic acid receptor *PYL4* (LOC107778387), auxin-responsive protein *IAA14* (LOC107764405), heat shock protein 90 (*Hsp90*, LOC107816677), WRKY transcription factor 40 (*WRKY40*, LOC107792337), WRKY transcription factor 50 (*WRKY50*, LOC107772027), serine/threonine-protein kinase *RLK1* (LOC107794887), pathogenesis-related protein-1C (*PR1*, LOC107763263) and TGACG-sequence-specific DNA-binding protein *TGA-2.1* (LOC107782457). The results showed that the expression levels of the auxin responsive genes were significantly decreased, whereas expression of other genes were increased in chimeras infected plants compared with TMV, which were consistent with the results of RNA-seq (Figure 4). Additionally, regression analyses showed that there was a positive correlation between RNA-seq and RT-qPCR data (Supplementary Figure S3).

### Host Defence Responsive Genes Induced by Infection of TMV-CGCP and TMV-PMCP

Plant virus infection often induces host-virus interactions with a strong plant immune response. Leucine-rich repeat LRR domain present in receptor-like kinases (RLKs) plays an important role in pathogen recognition through signaling pathways (Gouveia et al., 2016). In this study, we selected typical genes involved in plant-pathogen interaction and phytohormone signaling from the results of RNA-seq and listed their regulation after the chimeric virus infection (Table 2). We found several differentially expressed genes that belong to *RLK* gene-family were induced by chimeric virus infection (Table 2 and Supplementary Table S3). In addition, a variety of genes involved in host-pathogen interaction such as *FLS2*, brassinosteroid insensitive 1-associated receptor kinase 1 (*BAK1*), enhanced disease susceptibility 1 protein (*EDS1*) and heat shock proteins (*Hsps*) were significantly increased (Table 2). Infection of the TMV chimeras also induced differential expression of genes involved in immune signaling transduction such WRKY transcription factors, NAC transcriptional factors and mitogen-activated protein kinase kinase kinase (*MAPKKK*). Four DEGs including *RLK1*, *Hsp90*, *WRKY40* and *WRKY50* involved in defence response were selected to verify their regulation using RT-qPCR. The results demonstrated that the expression levels of all the above genes were significantly up-regulated by the infection of chimeras of TMV (Table 2). The results of RT-qPCR were consistent with that of RNA-seq (Figure 4 and Table 2).

**TABLE 2:** A general table showing critical DEGs involved in plant pathogen interaction and phytohormone signaling induced by TMV and the chimeras in *N. tabacum*.

Gene Symbol	Gene description*	Regulate	TMV vs. TMV- CGCP log_2_FC	TMV vs. TMV- PMCP log_2_FC	Gene functions	References
LOC107762702	regulatory protein NPR1	Up	1.84	1.69	salicylic acid receptors	Ding et al., 2018
LOC107782457	TGACG-sequence-specific DNA-binding protein TGA-2.1	Up	1.71	1.48	defense responses	Kegler et al., 2004
LOC107764405	auxin-responsive protein IAA14	Down	−1.83	−2.21	root development	Fukaki et al., 2005
LOC107780289	auxin-induced protein AUX22	Down	−1.29	−1.43	auxin-activated signaling pathway	
LOC107783912	auxin-responsive protein SAUR21	Down	−1.69	−1.93	auxin response	Spartz et al., 2012
LOC107808395	auxin-induced protein 15A	Down	−1.41	1.62	unknown function, auxin response?	
LOC107786234	two-component response regulator ARR9	Down	1.38	−1.64	cytokinin response	Ishida et al., 2008
LOC107778387	abscisic acid receptor PYL4-like	Up	3.06	3.41	ABA receptor	Lackman et al., 2011
LOC107819672	serine/threonine-protein kinase SRK2	Up	2.86	1.99	activate ABA-responsive genes	Fujii and Zhu, 2009
LOC107764158	DELLA protein GAI-like	Up	3.45	3.61	repressors of GA signal	Rodriguez et al., 2014
LOC107798341	gibberellin receptor GID1B-like	Up	4.85	4.06	GA perception, interact with DELLA protein	Hauvermale et al., 2014
LOC107774409	ABC transporter G family member 11	Up	6.63	4.12		
LOC107774521	ABC transporter A family member 11	Up	7.32	6.33	phytohormone transportation, plant growth and development	Do et al., 2018
LOC107798772	ABC transporter B family member 11	Up	3.25	4.57		
LOC107795861	EIN3-binding F-box protein 2-like	Up	1.11	1.19	regulating ethylene	Gagne et al., 2004
LOC107803442	WRKY transcription factor 26	Up	4.31	5.21	involved in ethylene-response signal transduction pathway	Li et al., 2011
LOC107811911	WRKY transcription factor 33	Up	3.17	3.46	positive regulator of salt stress response and ABA signaling	Jiang and Deyholos, 2009
LOC107792337	WRKY transcription factor 40	Up	8.82	9.07	regulate plant immunity, negatively regulate ABA	Liu et al., 2019
LOC107794887	serine/threonine-protein kinase RLK1	Up	6.26	5.46	pathogen recognition	Gouveia et al., 2016
LOC107773761	LRR receptor-like serine/threonine-protein kinase FLS2	Ups	7.992	5.771	PRR in PTI pathway	Garcia et al., 2014
LOC107794844	LRR receptor-like serine/threonine-protein kinase EFR	Up	4.14	3.02	PRR in PTI pathway	Zipfel et al., 2006
LOC107794142	mitogen-activated protein kinase kinase kinase YODA	Up	2.38	3.77	MAPK pathway	Sopena-Torres et al., 2018
LOC107790422	RPM1-interacting protein 4	Up	2.87	3.15	negatively regulate plant defense	Mackey et al., 2002
LOC107810911	pathogenesis-related genes transcriptional activator PTI6	Up	2.17	2.12	activate defense responses	Gu et al., 2002
LOC107811391	protein EDS1L	Up	2.05	1.58	regulate immune response	García et al., 2010
LOC107772027	WRKY transcription factor 50	Up	2.32	1.84	activate PR1	Hussain et al., 2018
LOC107773587	WRKY transcription factor 70	Up	4.97	7.74	regulate immune response	Pieterse et al., 2012
LOC107765311	RNA-dependent RNA polymerase 1	Up	1.76	2.12	basal resistance, antiviral RNA silencing	Qin et al., 2017
LOC107763938	TMV resistance protein N-like isoform X1	Up	1.77	1.74	confer resistance to TMV	Marathe et al., 2002
LOC107794263	wound-induced protein WIN1	Up	3.09	1.88	wound induced responses, anti-fungal activity	Ponstein et al., 1994
LOC107807832	pathogenesis-related protein 1B	Up	11.25	7.48	defense response	Breen et al., 2017
LOC107763263	pathogenesis-related protein 1C	Up	12.88	8.93	response to stress	
LOC107805462	respiratory burst oxidase homolog protein A	Up	2.27	1.32	response to stress	Wang et al., 2018
LOC107819672	serine/threonine-protein kinase SAPK1-like isoform X2	Up	2.86	1.99	response to salt stress	Lou et al., 2018
LOC107822929	heat shock 70 kDa protein	Up	1.08	1.14	biotic and abiotic stress response	Usman et al., 2017
LOC107816677	heat shock 90 kDa protein	Up	1.56	1.55	involved in ETI responses	Gouveia et al., 2016

*Genes selected for RT-qPCR verification and the model in Figure 7 were underlined.

### Differential Expression of Phytohormones and Their Responsive Genes Induced by Infection of the Chimeric TMV

The change of plant hormones is often accompanied by virus infection. Phytohormones play important roles in plant growth and development as well as host symptoms induced by virus infection (Collum and Culver, 2016). In this study, the transcriptome data indicated that various genes involved in hormone signaling pathways in TMV chimeras infected plants were differentially regulated compared with that of wild-type TMV (Figure 4 and Table 2). For SA signaling pathway, a variety of genes like non-expressor of pathogenesis-related genes 1 (*NPR1*), TGA Transcription Factor genes and pathogenesis-related protein-1 (*PR1*) were significantly increased upon chimeric TMV infection. By contrast, the expression of auxin signaling pathway-related genes including auxin transporter *AUX1*, *AUX/IAA* family and small auxin-up RNA (*SAUR*) were significantly decreased after infection of the chimeric viruses. In addition, expressions of ethylene (ET) related genes ethylene insensitive3 (*EIN3*) and ABA related genes including abscisic acid receptor *PYR/PYL* family and serine/threonine-protein kinase SRK2 (*SNRK2*) were induced in TMV-CGCP or TMV-PMCP infected plants. We selected *PYL4, PR1*, *TGA2.1*, and *IAA14* to verify their regulation using RT-qPCR. The results demonstrated that the expression levels of *PR1*, *TGA2.1*, and *PYL4* were significantly up-regulated and *IAA14* was significantly down-regulated by the infection of chimeric viruses (Figure 4). The results were consistent with that of RNA-seq (Figure 4 and Table 2).

**FIGURE 5:**
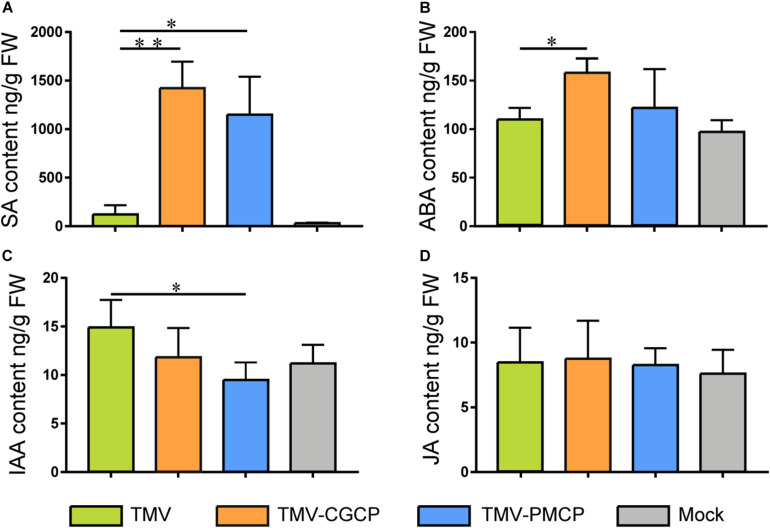
Phytohormone concentrations in *N. tabacum* infected with TMV, TMV-CGCP or TMV-PMCP. Mock-inoculated plants were used as control treatments (Mock). Contents of **(A)** SA, **(B)** IAA, **(C)** ABA and **(D)** JA in leaves of *N. tabacum* were determined at 10 dpi. Asterisks indicate a statistically significant difference compared with TMV, “^∗^” indicate a significant difference (*P* < 0.05) and “^∗∗^” indicate an extremely significant difference (*P* < 0.01).

Then, we determined the level of phytohormone including SA, IAA, ABA and JA by HPLC- MS. Mock-inoculated *N. tabacum* plants were used as control treatments. Our results showed that the SA level of *N. tabacum* inoculated with chimeras TMV-CGCP or TMV-PMCP was significantly higher than that in TMV or mock-inoculated plants, while the content of IAA in the chimeric virus inoculated plants exhibited obvious decrease than that in wild-type TMV inoculated plants (Figure 5). This result was parallel with the result of the transcriptome. Additionally, ABA level was significantly increased in TMV-CGCP inoculated plants, while ABA content in TMV-PMCP inoculated plants was similar to that of TMV inoculated or mock-inoculated plants (Figure 5). The levels of JA showed no significant difference among all of the virus inoculated plants (Figure 5).

### Alteration of Defence or Phytohormone Responsive Genes by Transient Expression of CP From Three Tobamoviruses

**FIGURE 6:**
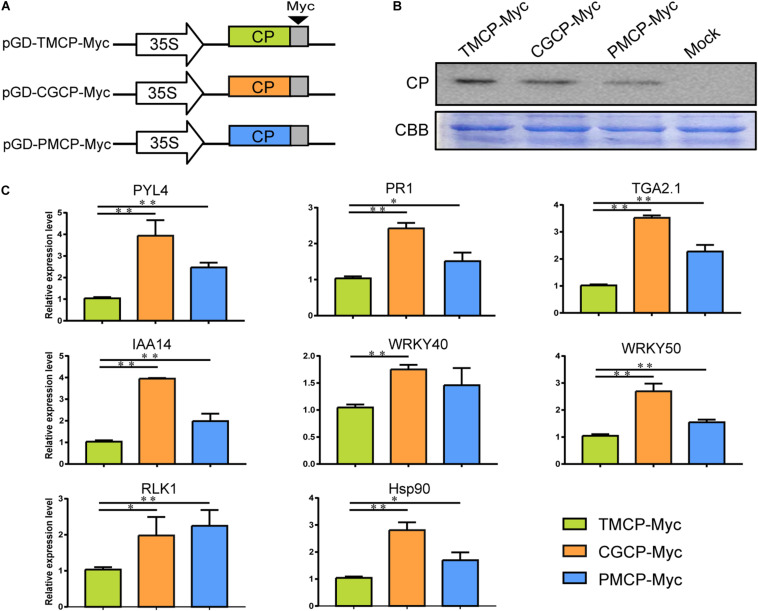
Expression of defence or phytohormone responsive genes regulated by transient expression of CP of TMV, CGMMP or PMMoV with Myc-tag in *N. benthamiana*. **(A)** Schematic representation of the transient CP expression vector pGD-TMCP-Myc, pGD-CGCP-Myc and pGD-PMCP-Myc. **(B)** Western blot analysis of the three tobamovirus CPs in leaves of *N. benthamiana* infiltrated with pGD-TMCP-Myc, pGD-CGCP-Myc and pGD-PMCP-Myc at 2 dpi. The Coomassie brilliant blue-stained cellular proteins were shown below the western blot as loading controls. **(C)** RT-qPCR analysis of relative expression levels of *PYL4*, *IAA14*, *Hsp90*, *WRKY40*, *WRKY50*, *RLK1*, *PR1*, and *TGA* in *N. benthamiana* leaves infiltrated with pGD-TMCP-Myc, pGD-CGCP-Myc or pGD-PMCP-Myc at 2 dpi. Information of the genes indicated in the figure were showed in Table 2. Asterisks indicate a statistically significant difference compared with TMV, “^∗^” indicate a significant difference (*P* < 0.05) and “^∗∗^” indicate an extremely significant difference (*P* < 0.01). For others, refer to the legends of Figure 5. Primers used for qPCR were listed in Supplementary Table S6.

To test if the CP from different tobamoviruses can induce differential expression of the defence or phytohormone responsive genes without viral genome RNA, we constructed three constructs (Figure 6A) and transiently express the Myc-tag fused coat protein of TMV (TMCP-Myc), CGMMV (CGCP-Myc), and PMMoV (PMCP-Myc) in *N. benthamiana* leaves, respectively. The results of western blot analysis showed that the accumulation levels of Myc-tag fused TMV, PMMoV, and CGMMV CP were similar (Figure 6B). Moreover, 8 genes corresponded with defence or phytohormone responses were selected for RT-qPCR verification and the results showed the significant up-regulation of *RLK1*, *Hsp90*, *PR1*, *PYL*, *IAA14*, *TGA*, *WRKY40*, and *WRKY50* induced by transient expression of CGCP-Myc or PMCP-Myc. The regulatory patterns of the gene expression were generally consistent with that induced by infection of TMV-CGCP or TMV-PMCP (Figure 4 and Figure 6C).

**FIGURE 7:**
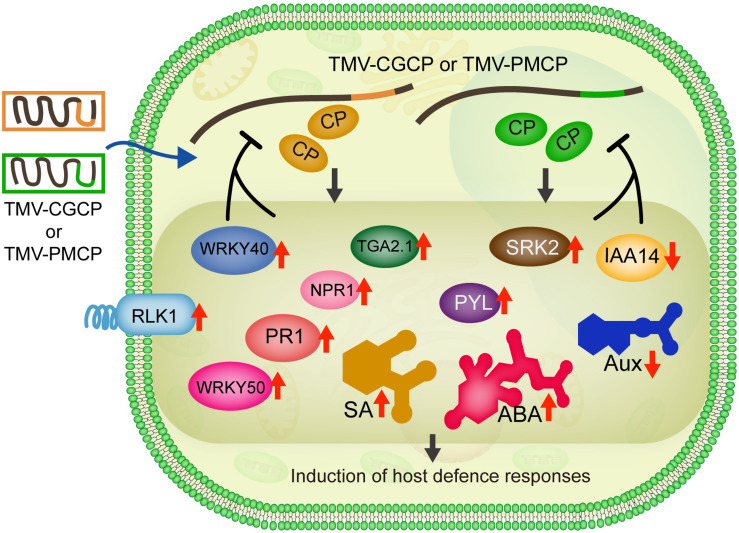
Model for gene and phytohormone regulation involved in induction of host defence responses by infection of chimeric TMV. Infection of TMV-CGCP as well as TMV-PMCP trigger the up-regulation of defence-responsive genes such as *RLK1*, *WRKY40* as well as genes involved in phytohormone signaling pathways (SA: *NPR1*, *TGA2.1*, *WRKY50* and *PR1*; ABA: *SRK2* and *PYL*) and induce increase in the levels of SA and ABA, but reduce the expression of *IAA14* and levels of Aux, respectively.

## Discussion

Application of chimeric viruses containing heterologous sequence substitution can provide important messages to clarify the critical functions of the RNA sequences or the coding proteins of plant viruses during infection (Yelina et al., 2002; Lukhovitskaya et al., 2013; Tuo et al., 2015; Yu et al., 2019). For example, chimeric barley stripe mosaic virus (BSMV) with exchange in γb protein from poa semilatent virus (PSLV) indicated RNA silencing suppression function of this viral protein. In addition, infection of chimeric TMV and potato virus X (PVX) expressing p12 of chrysanthemum virus B indicated that p12 plays an important role in modulation of host cell morphogenesis (Lukhovitskaya et al., 2013). Furthermore, a series of BSMV chimeras with exchanges in MP from various plant viruses revealed the functions of exogenous MP on the virus-coded transport and host symptoms (Solovyev et al., 1996, 1997, 1999). We previously also constructed various chimeric PMMoV clones to further elucidate the molecular mechanisms for the virus infection (Yu et al., 2019). In this work, chimeric virus TMV-CGCP and TMV-PMCP with heterologous tobamovirus coat proteins were constructed using homologous recombination technique, which greatly shortens the construction procedure as the unique restriction enzyme sites are not required to be introduced into the virus infectious clone.

To combat viral infection, plants have evolved sophisticated innate immune systems, which are generally divided into the PTI and ETI (Gouveia et al., 2016; Calil and Fontes, 2017). After recognition of invading pathogens by pattern recognition receptors (PRRs), the mitogen-activated protein kinases (MAPKs) cascades are rapidly activated and the transduction signal can eventually activate various WRKY transcription factors in plants (Gouveia et al., 2016). RLK1 is one of the well-conserved PRRs being critical for the perception of conserved microbe-associated molecular patterns (MAMPs) to activate PTI in different plant species (Hu et al., 2018). Notably, many studies have shown that WRKY transcription factors from various plant species are induced in response to viral infection (Huang et al., 2016; Huh et al., 2012). In this study, the results indicated that infection of TMV-CGCP or TMV-PMCP can induce rapid host defence responses by markedly up-regulating gene expression of *RLK1*, *WRKY40* and *WRKY50* compared with that of wild-type TMV infection. WRKY40 was reported to associate with a transcription factor BZR1 to mediate plant immune signaling (Lozano-Durán et al., 2013). In a previous study, we have also shown that *WRKY40* was up-regulated by an anti-viral biological agent Ningnanmycin (An et al., 2019), which also confirmed the function of WRKY40 in plant immune responses. It was revealed that *Arabidopsis* WRKY50 together with TGA transcription factors synergistically activate expression of *PR1* (Hussain et al., 2018), which were in agreement with our results showing that expression of *PR1* together with *WRKY50* were consistently up-regulated by infection of chimeric TMV. Plants can use elaborate strategies by inducing phytohormone-signaling networks to get through abiotic or biotic stresses, as well as inducing resistance response to pathogen infection (Calil and Fontes, 2017; Verma et al., 2016). In this study, the infection of TMV-CGCP and TMV-PMCP caused stunting in tobacco, which was possibly related to the significant changes in hormone expression.

The prominent contribution of ABA to plant defence response against abiotic stress conditions has long been investigated (Verma et al., 2016). In addition to its roles in development and abiotic stress responses, ABA also plays a multifaceted role in plant immunity (Ton et al., 2009). Additionally, ABA was shown to be involved in enhanced or reduced plant anti-viral responses (Alazem et al., 2017; Xie et al., 2018). ABA induce callose deposition at plasmodesmata (PD) as well as the RNA silencing pathway, which is proved to be involved in antiviral mechanisms (Alazem et al., 2017). Study has also shown a positive correlation between ABA and host resistance against TMV (Chen et al., 2013). In this study, our results showed that infection of the chimeric virus TMV-CGCP and TMV-PMCP induced significant increase of hormone ABA and ABA-responsive genes *PYL4* and *SRK2* in tobacco compared with that was infected by TMV. It was revealed that PYL4 acts as a receptor for ABA and is involved in plant metabolism and growth (Lackman et al., 2011). SNRK2s are a family of plant-specific protein kinases, which are activated by ABA and play an important role in the activation of ABA responsive genes (Fujii and Zhu, 2009). In this study, TMV-CGCP and TMV-PMCP induced significant increase of hormone ABA and ABA-responsive genes *PYL4* and *SRK2* in tobacco, which indicated that ABA signaling may be involved in the induced host resistance against the chimeric virus infection.

The increase in the levels of SA plays critical roles for host plants in resistance of a broad spectrum of pathogens such as biotrophic pathogens (or obligate parasites) when they trigger PTI or ETI responses. The SA is also widely mentioned in viral defence immunity (Conti et al., 2017). Exogenous SA treatment reduces TMV RNA accumulation by interfering with TMV replication in mesophyll cells and also inhibits cell to cell movement in epidermal cells (Chivasa et al., 1997; Murphy and Carr, 2002; Zhu et al., 2014). Moreover, an increase in SA levels in the pathogen-infected plant tissues results in the induction of *PR1*, which was considered as a defence marker gene (Breen et al., 2017). Therefore, the significant induction of *PR1* by infection of the chimeric TMV is consistent with the marked increase in SA levels after the chimeric TMV infection in tobacco. In addition, it was reported that TGA2.1 can interact with NPR1 and play crucial roles in SA induction (Thurow et al., 2005). Therefore, the significant induction of *PR1* and *TGA2.1* is consistent with the marked increase of SA levels after the chimeric virus infection in tobacco.

SA regulates plant basal defences via activating the host systemic acquired resistance (SAR) and hypersensitive responses. Induction of SAR also suppresses a serial of auxin-responsive genes (Kovtun et al., 1998), suggesting that enhanced resistance to diseases would necessitate repression of auxin signaling (Verma et al., 2016). A previous study showed that TMV p126 replicase interacts with a variety of Aux/IAA proteins to influence the expression of a large number of auxin responsive genes and induce specific disease symptoms (Padmanabhan et al., 2005). In general, the SA and JA signaling pathways influence each other through a complex network of antagonistic interactions (Koornneef and Pieterse, 2008). JA, a kind of plant hormone, is mainly responsible for defence against necrotrophic pathogens and herbivorous insects (Wasternack and Hause, 2013). A study had indicated that SA as well as JA are required for systemic response to TMV (Zhu et al., 2014). In this study, SA was rapidly induced by infection of TMV-CGCP or TMV-PMCP than TMV, whereas the JA accumulation remained consistent after TMV or the chimeric TMV infection. The results indicated that the significant increase of SA after the chimeric TMV infection may be responsible for the stunting and necrotic symptoms in tobacco.

Viral CP is crucial for viral RNA encapsidation to form virions, which can protect the virus from nuclease degradation and RNA silencing of the host plants (Ivanov and Mäkinen, 2012). The CP also plays important roles in the infection, proliferation, systemic movement, pathogenicity and transmission of the virus (Bendahmane et al., 2002; Conti et al., 2017). CP is also closely involved in the disease symptomology of the virus-infected plants. For cucumber mosaic virus (CMV), single amino acid substitutions in the CP ORF affected the thylakoid structure of tobacco chloroplasts (Mochizuki and Ohki, 2011). Additionally, the *N. benthamiana* NbPCIP1 was found to interact with the CP of PVX, which affect infectivity, pathogenicity, and symptom expression of the virus (Park et al., 2009). A previous study showed that TMV with truncate CP induced stunt and necrosis symptoms in tobacco (Kurihara and Watanabe, 2004), which are very similar to the symptoms caused by TMV-CGCP and TMV-PMCP. By comparing the CP amino acid sequences of TMV, CGMMV and PMMoV, we found that the amino acid similarity of TMV CP and CGMMV CP was approximately 35%, whereas the amino acids of PMMoV CP are more closely related with that of TMV with the similarity of 70% (Data not shown). It was reported that an amino acid substitution introduced into CP ORF (CP^T42W^) can restrict the accumulation of MP and result in strong resistance to TMV infection in plants containing CP^T42W^ (Bendahmane et al., 2002). In this study, infection of TMV-CGCP and TMV-PMCP showed reduced viral RNA accumulation compared with wild-type TMV, which possibly due to the induced resistance of the plants caused by the respective CPs. Furthermore, the transiently expressed CP exhibit similar regulatory effect on host gene expression. Therefore, it is possible that free CP subunit of CGMMV or PMMoV also affect host transcriptome as well as the chimera virions. Our results also indicated that the exogenous substituted CP does not affect the ability of virion assembly of TMV-CGCP and TMV-PMCP despite the low sequence and amino acid similarity. Such results also demonstrated that the CP of CGMMV or PMMoV can recognize RNA sequences of TMV and subsequently perform virion assembly.

In the previous work, we have showed that CP defective virus PMMoV-fsCP can systemically infected *N. benthamiana* and *C. annuum* and confirmed the introduced mutation was maintained in the systemic leaves of the inoculated plants (Yu et al., 2019). In this study, the CP defective viruses TMV-fsCP, TMV-CGfsCP and TMV-PMfsCP can systemically infect tobacco but do not induce observable symptoms. These data indicated that CP is dispensable for systemic movement of TMV, but it may contribute to increasing the infection efficiency and play important roles in symptom development.

Normally, viruses do not induce the death of plants in the infection defence equilibrium. Some plant virus even has RNA silencer elements in its genome RNA to suppress the virus from too fast replication (Pogany et al., 2003), which better facilitate the virus to survive and spread. Therefore, the reduction of the adverse effects of the virus infection in host plants is beneficial for the transmission, survival and evolution of the viruses. These chimeric viruses expressing coat proteins from CGMMV or PMMoV may affect host metabolic pathways or affect the interactions between other viral proteins and the host proteins, thus leading to the significant defence responses of tobacco. To clarify the statements, further investigations are required to determine the critical domain or amino acids of coat protein as well as other viral proteins of TMV. We collectively summarized our data in Figure 7. Based on these results, we also speculate that the CP of wild-type TMV may elaborately suppress or evade the host defence machinery of tobacco to reduce the adverse impact on the plant, which should be due to the results of long-period of sophisticated co-evolution between plant viruses and their hosts.

## Data Availability Statement

The datasets presented in this study can be found in online repositories. The names of the repository/repositories and accession number(s) can be found in the article/Supplementary Material.

## Author Contributions

YW conceived the research project. MA, ZX, MY, and XB conceived and designed the experiments. MY, XB, and YH performed the main experiments. YC, JW, RZ, and YL cultivated *N. benthamiana* plants and *N. tabacum* cv and NC89 and treated samples for transcriptome sequencing. MA, MY, and XB carried out the transcriptome analysis and analyzed the data. YW, MA, ZX, MY, and XB prepared the reagents and materials and analysis tools. MA and MY prepared the figures and tables. MY wrote the original draft. YW, MA, ZX, and XB reviewed the drafts of the manuscript. All authors reviewed and approved the manuscript.

## Conflict of Interest

The authors declare that the research was conducted in the absence of any commercial or financial relationships that could be construed as a potential conflict of interest.

## Footnotes

1 http://www.ncbi.nlm.nih.gov/Traces/sra

2 http://ccb.jhu.edu/software/hisat2/index.shtml

3 http://www.bioconductor.org/packages/release/bioc/html/DESeq.html

4 http://www.genome.jp/kegg/

## Supplementary Material

Supplementary Figure 1 Construction of chimeras TMV-fsCP, TMV-CGfsCP and TMV-PMfsCP and their symptoms and accumulation in *N. tabacum*. **(A)** Schematic representation of genome structure of the construct TMV-fsCP, TMV-CGfsCP and TMV-PMfsCP, site mutation was introduced to the start codon of the CP based on TMV, TMV-CGCP and TMV-PMCP, respectively. **(B)** Symptoms induced in *N. tabacum* by TMV-fsCP, TMV-CGfsCP and TMV-PMfsCP at 7 dpi. Mock-inoculated plants were used as control treatments (Mock). **(C)** Relative accumulation of virus in *N. tabacum* inoculated with TMV-fsCP, TMV-CGfsCP and TMV-PMfsCP at 7 dp, compared to TMV, TMV-CGMP and TMV-PMCP, respectively. Asterisks indicate a statistically significant difference compared with TMV, “^∗^” indicate a significant difference (*P* < 0.05) and “^∗∗^” indicate an extremely significant difference (*P* < 0.01).
