# Supplementary figures and images for: Chimeric Tobamoviruses With Coat Protein Exchanges Modulate Symptom Expression and Defence Responses in Nicotiana tabacum
Source: Front Microbiol. 2020 Nov 6;11:587005. doi: 10.3389/fmicb.2020.587005 (PMC7677242; doi:10.3389/fmicb.2020.587005)

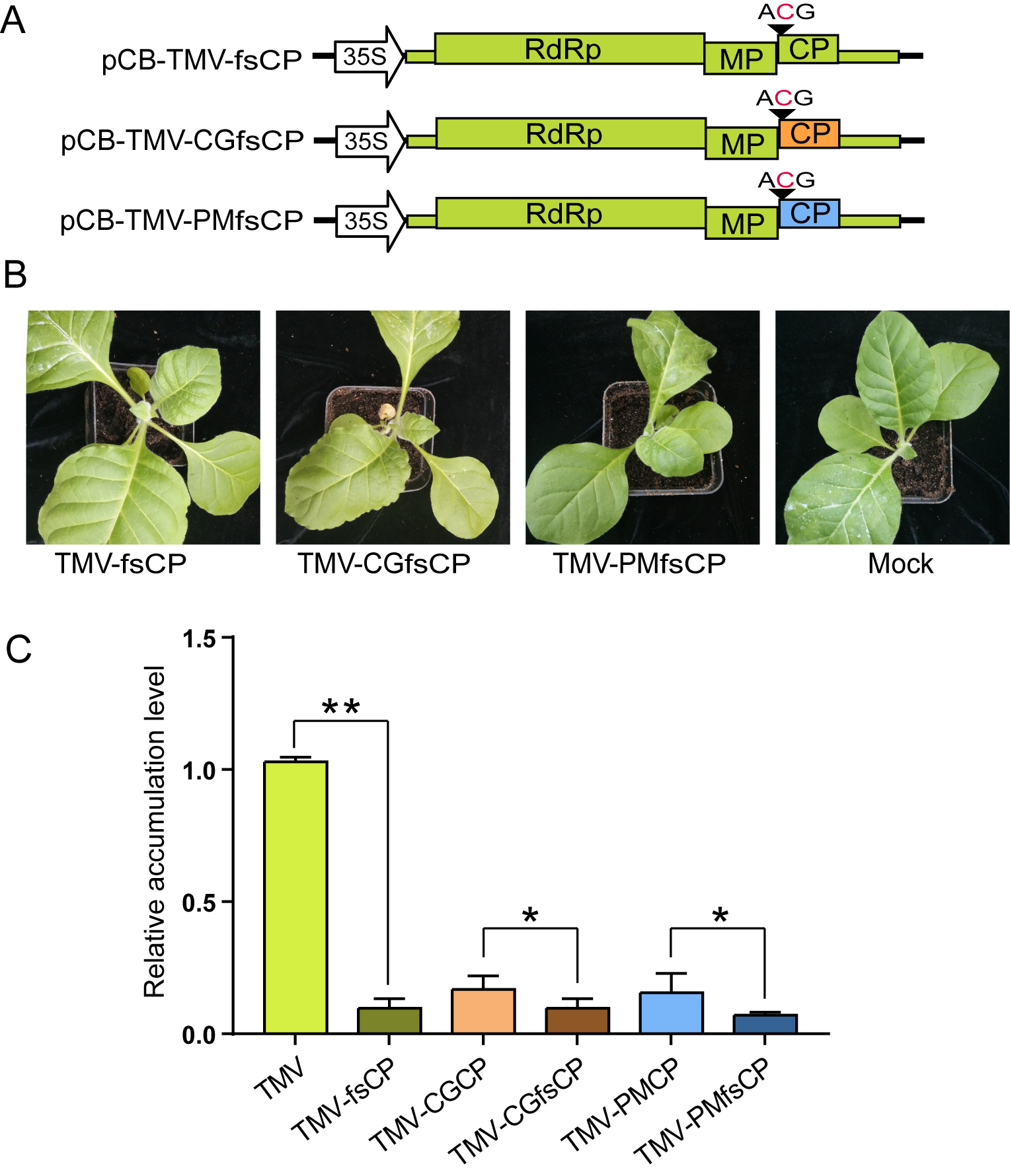

Supplement: Supplementary Figure 1 — Construction of chimeras TMV-fsCP, TMV-CGfsCP and TMV-PMfsCP and their symptoms and accumulation in N. tabacum. (A) Schematic representation of genome structure of the construct TMV-fsCP, TMV-CGfsCP and TMV-PMfsCP, site mutation was introduced to the start codon of the CP based on TMV, TMV-CGCP and TMV-PMCP, respectively. (B) Symptoms induced in N. tabacum by TMV-fsCP, TMV-CGfsCP and TMV-PMfsCP at 7 dpi. Mock-inoculated plants were used as control treatments (Mock). (C) Relative accumulation of virus in N. tabacum inoculated with TMV-fsCP, TMV-CGfsCP and TMV-PMfsCP at 7 dp, compared to TMV, TMV-CGMP and TMV-PMCP, respectively. Asterisks indicate a statistically significant difference compared with TMV, “∗” indicate a significant difference (P < 0.05) and “∗∗” indicate an extremely significant difference (P < 0.01). [file Image_1.TIF]

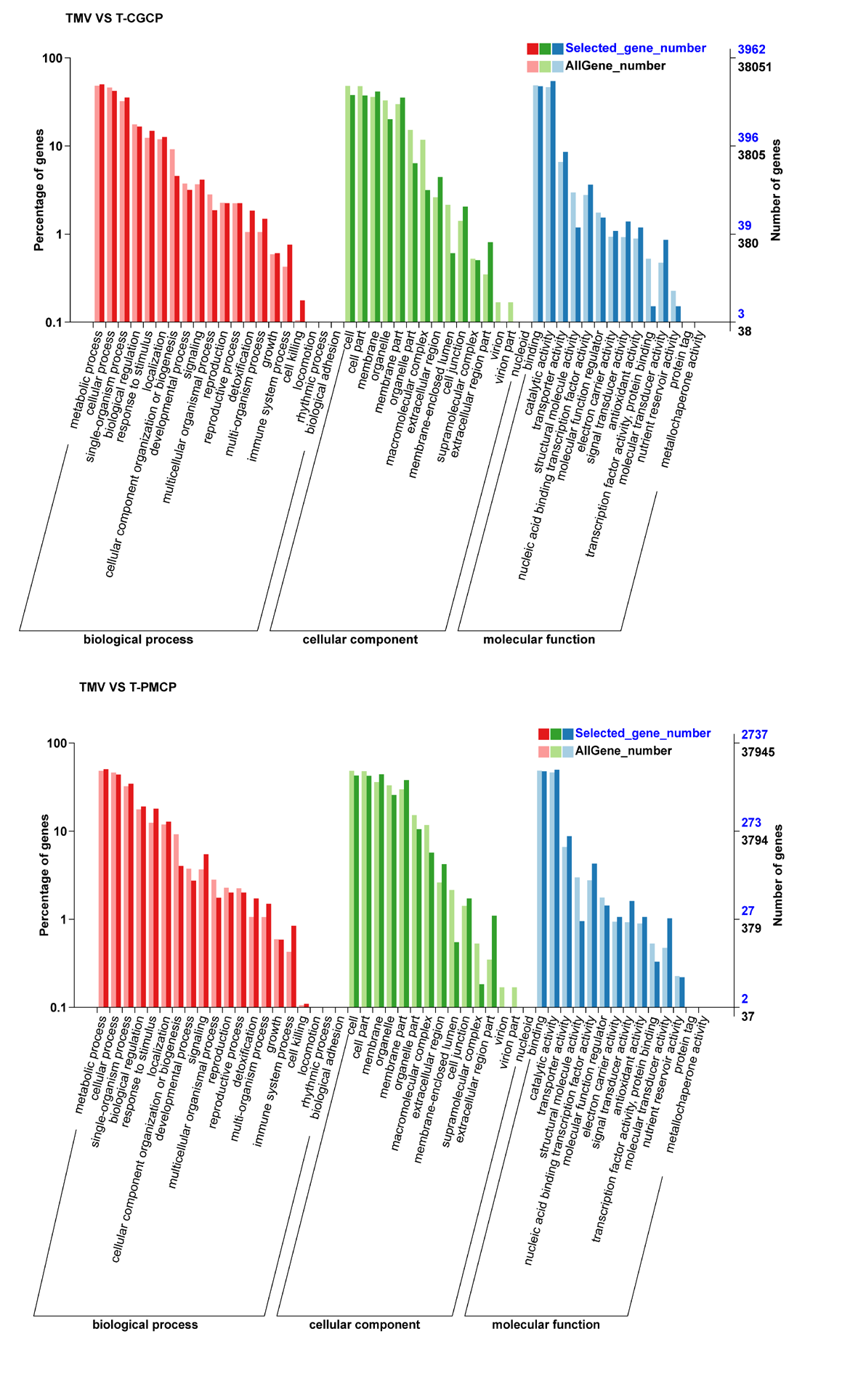

Supplement: Supplementary file 2 [file Image_2.TIF]

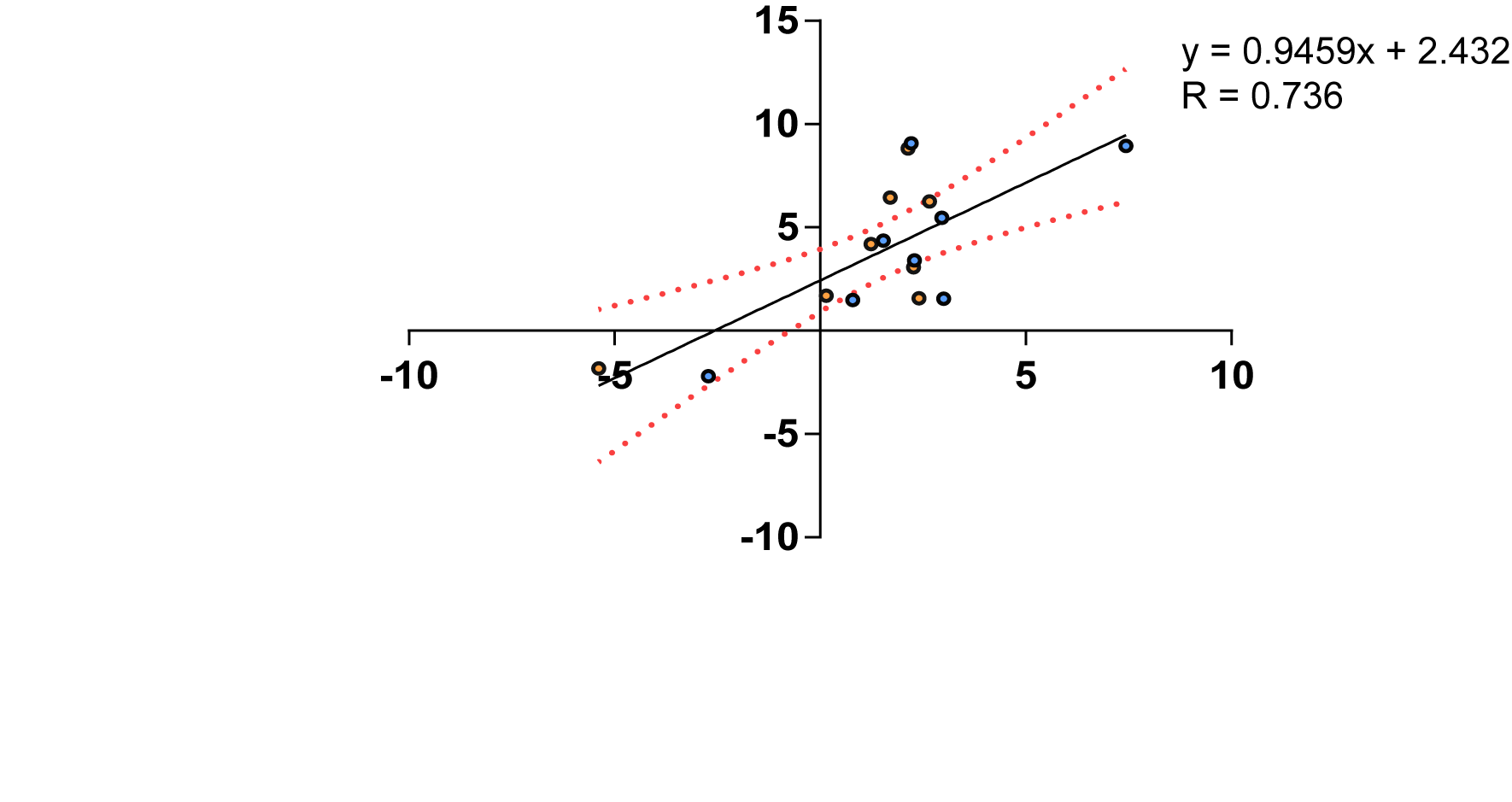

Supplement: Supplementary file 3 [file Image_3.TIF]
