# Supplementary material for: Chimeric Tobamoviruses With Coat Protein Exchanges Modulate Symptom Expression and Defence Responses in Nicotiana tabacum
Source: Front Microbiol. 2020 Nov 6;11:587005. doi: 10.3389/fmicb.2020.587005 (PMC7677242; doi:10.3389/fmicb.2020.587005)
Supplement: Supplementary file 10 [file Data_Sheet_1.PDF]

Supplementary Materials

Figure S1. Symptoms induced by TMV-fsCP, TMV-CGfsCP and TMV-PMfsCP and their relative accumulation compared to TMV, TMV-CGCP and TMV-PMCP, respectively, in *N. tabacum*. (A) Schematic representation of genome structure of pCB-TMV-fsCP, pCB-TMV-CGfsCP and pCB-TMV-PMfsCP, site mutation was introduced to the CP start codon of TMV, TMV-CGCP and TMV-PMCP, respectively. (B) Symptoms induced in *N. tabacum* by TMV-fsCP, TMV-CGfsCP and TMV-PMfsCP at 7 dpi. Mock-inoculated plants were used as control treatments (Mock). (C) Relative accumulation of virus in *N. tabacum* inoculated with TMV-fsCP, TMV-CGfsCP and TMV-PMfsCP at 7 dpi, compared to TMV, TMV-CGCP and TMV-PMCP, respectively. Asterisks indicate a statistically significant difference compared with TMV, "*" indicate a significant difference ($p < 0.05$) and "**" indicate an extremely significant difference ($P < 0.01$).

Figure S2. Gene Ontology (GO) terms of three categories significantly enriched in DEGs of TMV vs TMV-CGCP and TMV vs TMV-PMCP involved in molecular function, cellular component and biological process.

Figure S3. Regression analysis of RNA-seq (independent variable) and RT-qPCR (dependent variable) data. A regression analysis was performed to evaluate correlation between RNA-seq and RT-qPCR data. Orange dots represent plants inoculated with TMV-CGCP and blue dots represent plants inoculated with TMV-PMCP. Regression analyses were carried out in GraphPad Prism 7.

Table S1. Nucleic acid sequences of oligonucleotide primers used for vector construction and RT-PCR.
Table S2. Nucleic acid sequences of oligonucleotide primers used to qPCR analysis.
Table S3. Total DEGs in TMV vs TMV-CGCP and TMV vs TMV-PMCP.
Table S4. GO enrichment of DEGs in TMV vs TMV-CGCP and TMV vs TMV-PMCP.
Table S5. List of DEGs involved in the KEGG pathways in TMV vs TMV-CGCP and TMV vs TMV-PMCP.
Table S6. Total annotation of KEGGs in TMV vs TMV-CGCP and TMV vs TMV-PMCP.
